# The Application of Root Mean Square Electrocardiography (RMS ECG) for the Detection of Acquired and Congenital Long QT Syndrome

**DOI:** 10.1371/journal.pone.0085689

**Published:** 2014-01-15

**Authors:** Robert L. Lux, Christopher Todd Sower, Nancy Allen, Susan P. Etheridge, Martin Tristani-Firouzi, Elizabeth V. Saarel

**Affiliations:** 1 Nora Eccles Harrison Cardiovascular Research and Training Institute, University of Utah, Salt Lake City, Utah, United States of America; 2 The Division of Pediatric Cardiology, University of Utah, Salt Lake City, Utah, United States of America; University of Adelaide, Australia

## Abstract

**Background:**

Precise measurement of the QT interval is often hampered by difficulty determining the end of the low amplitude T wave. Root mean square electrocardiography (RMS ECG) provides a novel alternative measure of ventricular repolarization. Experimental data have shown that the interval between the RMS ECG QRS and T wave peaks (RT_PK_) closely reflects the mean ventricular action potential duration while the RMS T wave width (TW) tracks the dispersion of repolarization timing. Here, we tested the precision of RMS ECG to assess ventricular repolarization in humans in the setting of drug-induced and congenital Long QT Syndrome (LQTS).

**Methods:**

RMS ECG signals were derived from high-resolution 24 hour Holter monitor recordings from 68 subjects after receiving placebo and moxifloxacin and from standard 12 lead ECGs obtained in 97 subjects with LQTS and 97 age- and sex-matched controls. RT_PK_, QT_RMS_ and RMS TW intervals were automatically measured using custom software and compared to traditional QT measures using lead II.

**Results:**

All measures of repolarization were prolonged during moxifloxacin administration and in LQTS subjects, but the variance of RMS intervals was significantly smaller than traditional lead II measurements. TW was prolonged during moxifloxacin and in subjects with LQT-2, but not LQT-1 or LQT-3.

**Conclusion:**

These data validate the application of RMS ECG for the detection of drug-induced and congenital LQTS. RMS ECG measurements are more precise than the current standard of care lead II measurements.

## Introduction

The primary clinical assessment of ventricular repolarization involves measurement of the QT interval on the surface ECG. Prolonged ventricular repolarization is a known risk factor for *torsades de pointes* arrhythmia and sudden cardiac death. Precise measurement of QT interval is of paramount importance for the correct diagnosis of Long QT Syndrome (LQTS) and for the proper safety assessment of drugs in development. However, the QT is a low signal-to-noise measurement and the end of T wave can be difficult to determine in the presence of noise or encroaching P or U waves. Measurement of QT intervals are technically difficult in many patients with chronic heart disease whose T-waves often have low amplitude. In particular, P waves frequently encroach on the T wave downslope in newborns whose cycle lengths (CL) are much shorter than those in older children or adults. Thus, there are practical and clinically relevant reasons to consider alternative measures of ventricular repolarization.

One such alternative measure of repolarization makes use of the RMS ECG signal, a magnitude function of the ECG from which robust beat-to-beat measures of repolarization can be assessed [1]. The RMS ECG was classically used to define the *spatial magnitude* of the vectorcardiogram and to delineate the peak, onset and offset of the P, QRS and T wave signals. Likewise, the spatial magnitude of the standard 12-lead ECG can be calculated as follows: 
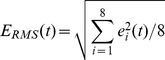
(Eq. 1)where *e_i_(t)* is the ECG signal at time *t* from lead *i*. Note that there are only eight independent signals of the twelve lead ECG. RMS ECG derived measurements have been experimentally validated using floating micro electrodes and refractory periods as estimates of mean times of ventricular depolarization and repolarization, mean ventricular action potential duration (APD), and dispersion of repolarization times [2]–[5]. Specifically, the R peak to T peak (RT_PK_) interval of the RMS ECG provides a robust estimate of the mean ventricular APD [2]–[5]. While substantial experimental data are available from animal studies, validation of RMS electrocardiography in humans is lacking. Thus, the objective of this study was to validate the application of RMS electrocardiography to detect abnormal ventricular repolarization in human subjects with drug-induced repolarization changes or congenital LQTS and compare the precision to that of current standard-of-care Lead II QT measurements.

## Methods

### Human Subjects and ECG Recordings

All experimental procedures performed in this study were approved by the University of Utah and Primary Children's Medical Center Institutional Review Boards (IRBs). Analysis of de-identified 12 lead ECG data from LQTS subjects and de-identified digital Holter data from healthy subjects were performed under a waiver of authorization of consent that was approved by the University of Utah and Primary Children's Medical Center IRBs. 24-hour Holter studies were performed on a limited number of LQTS pediatric subjects, after obtaining parental permission and assent permission that were documented in writing. Parental permission and assent documents were approved by the University of Utah and Primary Children's Medical Center IRBs.

De-identified data from a published placebo-controlled moxifloxacin thorough QT (TQT) study were obtained from The Telemetric and Holter ECG Warehouse (THEW) initiative (details at http://www.thew-project.org/) [6]. Briefly, 68 subjects (40 men, 28 women: 20–59 years) underwent two 24-hour continuous 12-lead digital ECG monitoring sessions (1000 Hz sampling rate using H12+ Holter monitors, Mortara Instruments, Milwaukee, WI) during randomized and blinded administration of placebo and a single 400 mg moxifloxacin dose. Raw 10 second, 12-lead ECGs from LQTS patients and healthy controls ascertained in the University of Utah Pediatric Cardiology clinic and Primary Children's Medical Center, Salt Lake City, Utah were identified and extracted from a GE Muse system. Healthy controls consisted of patients who were evaluated by a pediatric cardiologist and diagnosed with an innocent murmur and a normal 12-lead ECG. Ninety-seven LQTS patients (mean age 9.5±6.7 years) and 97 sex-matched controls (mean age 9.4±6.5 years) were identified. In 62 LQTS patients a mutation was identified in one of the major LQTS genes (LQT-1: 37 subjects, LQT-2: 18 subjects, LQT-3: 7 subjects). The remaining 35 patients manifested overt LQTS, but the genotype was negative or unknown.

### ECG analysis

**Figure 1:**
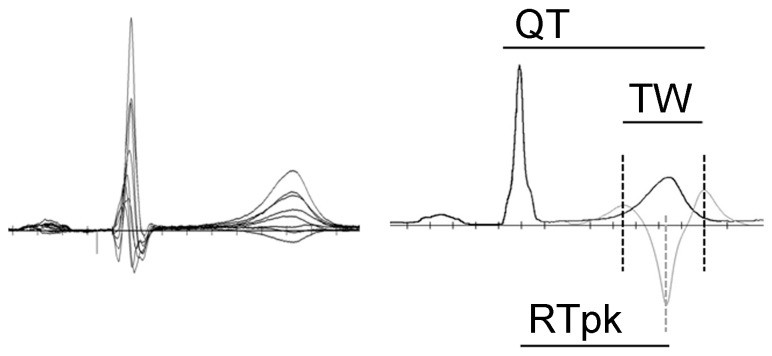
Determination of the RMS ECG signal. **Left panel**, Superimposition of 12-lead ECG traces corresponding to a single, exemplar beat obtained from a 24 hr Holter recording. **Right panel**, RMS signal derived from the single beat in left panel. Light grey trace represents the curvature index[2]–[5] of the RMS ECG signal, from which QT_RMS_, RT_PK_ and TW intervals were determined.

Raw 24 hour Mortara H12+ and 10 second GE Muse recordings were analyzed using in-house software (ScalDyn) to measure CL, RT_pk_, QT_RMS_ using a curvature index to identify T_pk_ and T_end_ [2] (Fig. 1) and Lead II QT (QT_II_) using the conventional *tangent method*. Non sinus rhythm beats and beats with excessive noise were excluded from analysis.

### Data and statistical analysis

For 24-hour recordings, QT_RMS_, RT_PK_, RMS TW, QT_II_ and CL were determined on a beat-beat basis and mean and standard deviations (STD) for each 10-minute epoch were calculated. Importantly, STDs include measurement variation as well as CL-dependent repolarization variation for each ten-minute epoch. Beats having CL outside the range (400–1200 ms) were excluded, as were noisy or non sinus rhythm adjacent beats. For 24 hour recordings, average QT_RMS_, RT_PK_ and conventional QT_II_ measurements were rate-corrected in a patient-specific manner using a linear regression model, as this was determined to be the simplest best fit.

Statistical analysis was performed using a hierarchical linear mixed model (Systat V.13, Systat Inc, Chicago, IL). Fixed effects included treatment (placebo vs moxifloxacin) crossed with time following placebo or moxifloxacin administration. The individual patient was included as a random effect. The Fisher's Least Significant Difference test was used to determine individual differences between groups. A p value of <0.05 was considered to be significant. All data are presented as mean ± SD.

## Results

### Application of RMS electrocardiography in drug-induced prolongation of ventricular repolarization

**Figure 2:**
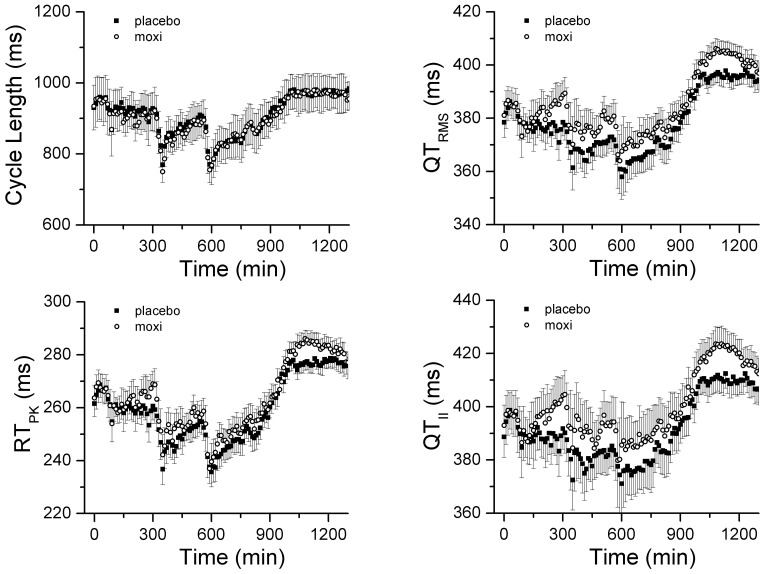
Distribution of RMS ECG intervals throughout the recording period. The time course of changes in RMS ECG parameters including cycle length, CL, RT_PK_, QT_RMS_, and QT_II_ intervals is presented. Data points represent 10 min averages (mean ± STD) from 68 subjects following placebo (filled square) or moxifloxacin (open circles) administration. Arrow indicates time point of placebo or moxifloxacin administration.

Consistent diurnal variations in CL were observed throughout the study duration for all subjects, with reductions in CL associated with study procedures and increases in CL related to sleep (Fig. 2). The relationships between QT_RMS_, RT_PK_ and QT_II_ intervals during the study periods are also illustrated in Fig. 2. QT_RMS_, RT_PK_ and QT_II_ intervals began to rise within 70 minutes of moxifloxacin administration and achieved their peak prolongation at 150 minutes post-moxiflaxacin. No changes in these intervals were observed during the comparable time period following placebo administration. Using a hierarchical linear mixed model, the uncorrected QT_RMS_, RT_PK_ and QT_II_ intervals were significantly different between placebo and moxifloxacin treatment groups, over the recording time period (Fig 2B, C and D; all p<0.0001), consistent with other published TQT studies [7]–[9].

**Figure 3:**
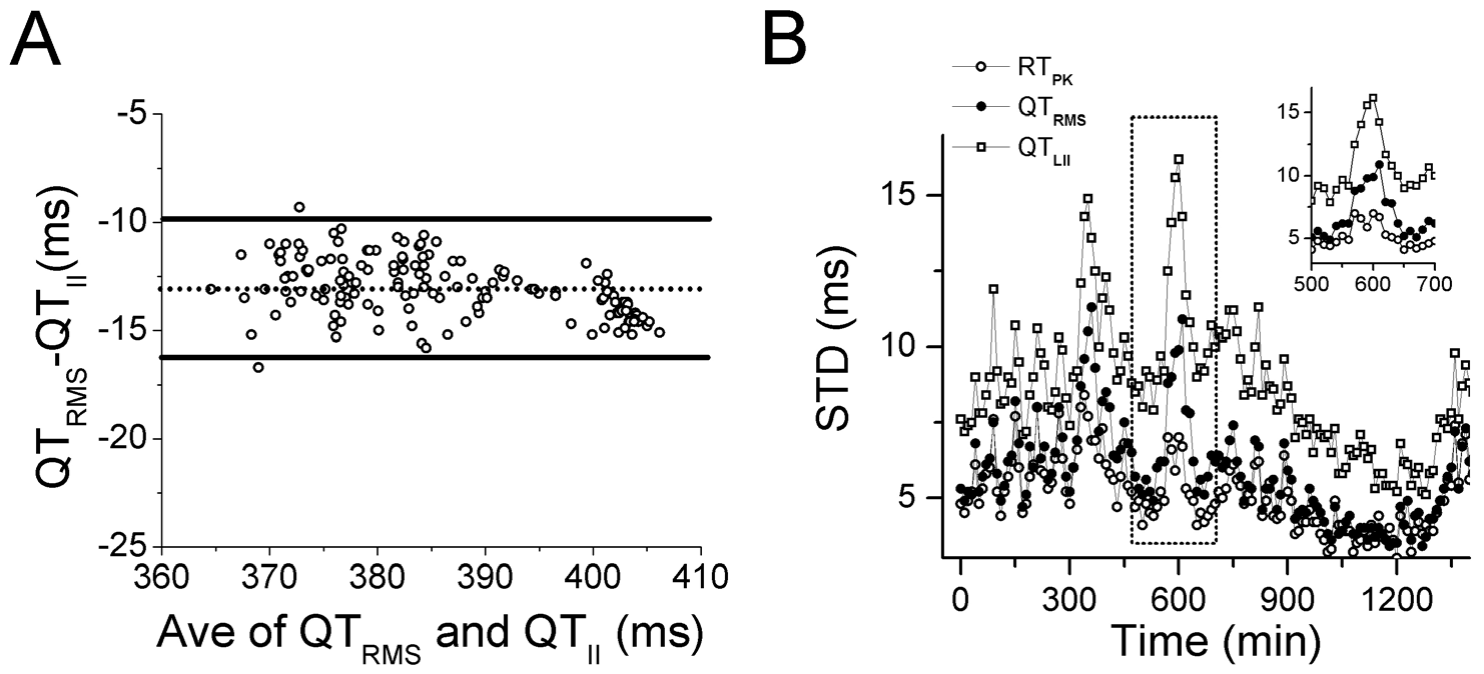
Direct comparison of repolarization measures. **Panel A**, Bland-Altman plot comparing QT_RMS_ and QT_II_ intervals. The dashed line represents the mean difference between QT_RMS_ and QT_II_ intervals (13 ms). The solid lines represent the 95% confidence intervals (±3 ms). The narrow confidence intervals indicate that the two methods are essentially equivalent; that is, both measures track the same repolarization phenomenon. **Panel B**, Mean standard deviations of RT_PK_, QT_RMS_ and QT_II_ intervals versus recording time. Inset highlights differences between the measures at a time of rapid change in CL (delineated by dotted line box).

In order to determine the level of agreement between the standard-of-care QT_II_ intervals and the QT_RMS_, we used a Bland-Altman analysis, whereby the differences between the two measures are plotted against the averages of the two measures [10]. The Bland-Altman plot (Fig. 3A) reveals that the QT interval measured by standard Lead II tangent method is ∼13 ms longer than the RMS derived measure, indicating that the end of the T wave is determined differently for each measure. However, the narrow confidence intervals (95%CI ± 3 ms) indicate that the two methods are essentially equivalent; that is, both measures track the same repolarization phenomenon.

Next, we sought to determine the intrinsic variability of the RMS measurements compared to the current standard-of-care QT_II_, by measuring the STD in each subject for each 10 min epoch. Over the entire placebo study period, the STD of QT_II_ was greater than QT_RMS_ and RT_PK_ values (Fig. 3B). Moreover, 2 spikes were observed for the QT_II_ and QT_RMS_ STD (360 and 580 min) that corresponded to abrupt decreases in CL (see Fig. 2). By contrast, RT_PK_ STD varied little in response to the same changes in CL (Fig. 3B, inset). Based on the smaller variance, the QT_RMS_ and RT_PK_ represent a more robust measure of repolarization duration than the standard QT_II_ measurement.

**Figure 4:**
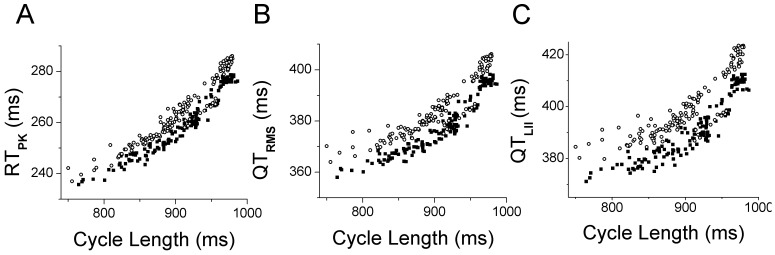
Relationships between mean RT_PK_, QT_RMS_ and QT_II_ intervals and mean cycle length. The 10 minute epoch mean QT_RMS_, RT_PK_, and QT_II_ intervals for 68 subjects are plotted versus corresponding mean CL for placebo (filled square) and moxifloxacin (open circles) treatments.

**Figure 5:**
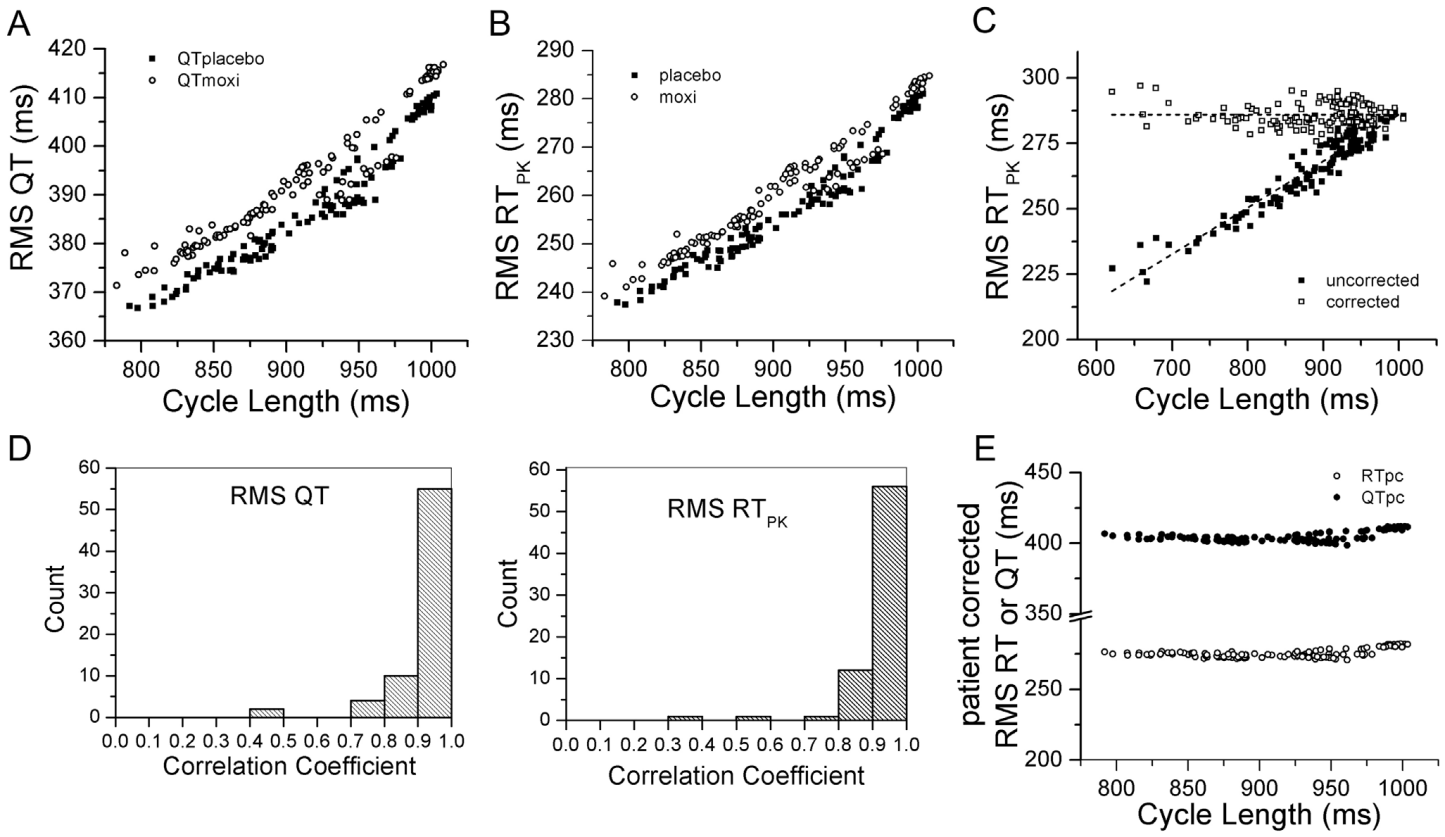
Heart rate correction. 10-min mean RT_PK_ values plotted versus mean CL for a single, representative subject in the placebo arm (**Left Panel**). RT_PK_ and QT_RMS_ values for this subject were corrected by applying a linear fit to the uncorrected ten minute averages of RT_PK_ versus cycle length relationship (**Right Panel**). The slope of the corrected RT_PK_ versus cycle length relationship was −0.00002. Similar results were obtained for all other subjects in both placebo and moxifloxacin treatment arms.

The relationships between the mean QT_RMS_ RT_PK_ and QT_II_ intervals and mean CL are shown in Fig. 4. Moxifloxacin administration shifted the relationships upward compared to placebo administration (all p<0.05), consistent with prolongation of ventricular repolarization. Next, we determined the best method of patient-specific heart rate correction, by plotting 10-minute average QT_RMS_ and RT_PK_ intervals versus CL for each individual patient. A representative relationship for one subject is displayed in Fig. 5, together with heart rate correction using a linear fit. The Pearson's correlation coefficient value for a linear regression fit of uncorrected values was 0.96 for this patient. After correcting for heart rate using a linear regression, the RT_PK_ vs CL relationship was essentially flat with a slope of −0.00002. For a majority of cases, the relationships between mean QT_RMS_ and RT_PK_ intervals versus mean CL were best fit by a linear regression model. Of note was the fact that rate dependency of QT_RMS_ and RT_PK_ *within* 10-minute epochs was minimal (regression slopes ∼0.02). The linear regression patient-specific heart rate correction was found to be superior to either parabolic or Fridericia heart rate corrections (data not shown).

**Figure 6:**
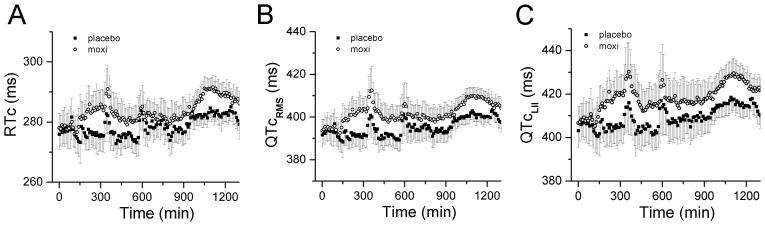
Heart rate-corrected QT_RMS_, RT_PK_ and QT_II_ intervals and moxifloxacin treatment. The time course of changes in heart rate-corrected QT_RMS_ (**Panel A**) and RT_PK_ (**Panel B**) intervals are presented for placebo (filled square) and moxifloxacin (open circles) treatments, using patient-specific, linear heart rate correction as in Fig 5.

**Figure 7:**
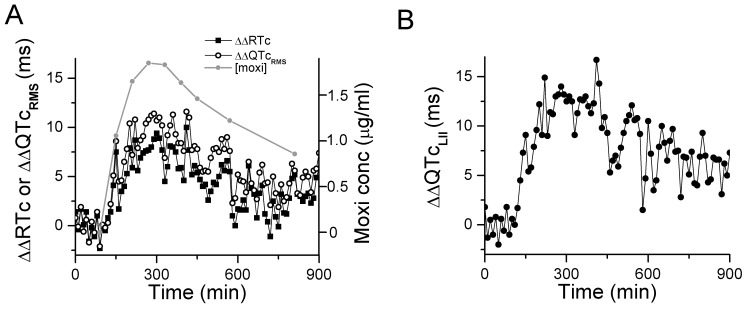
Baseline corrected and control corrected patient-specific heart rate-corrected mean values. QT_RMS_, RT_PK_ (**Left Pan**el) and QT_II_ (**Right Panel**) intervals plotted versus time. Also plotted is the moxifloxacin plasma concentration. The time course of changes in delta-delta QT_RMS_, RT_PK_ and QT_II_ intervals track that of changes in plasma moxifloxacin levels.

Mean and STDs of RT_PK_, QT_RMS_ and QT_II_ values, adjusted for mean CL using patient-specific linear correction for the placebo and moxifloxacin study periods are displayed in Fig. 6. The heart rate corrected values for each of these variables were significantly different between placebo and moxifloxacin groups (all p<0.0001). The patient-specific heart rate corrected placebo values were corrected from baseline and subtracted from the moxifloxacin values, so called *delta-delta* (Δ ΔQT_RMS_, RT_PK_ and QT_II_), and plotted together with mean moxifloxacin plasma concentration (Fig. 7). The peak Δ ΔQT_RMS_, Δ ΔRT_PK_ and Δ ΔQT_II_ values corresponded to the peak moxifloxacin plasma levels, occurring approximately 3 hours after moxifloxacin administration. The magnitude of the moxifloxacin-induced delta-delta change in QT_RMS_ was 11.5 ms compared to 9.5 ms for the RT_PK_ value. Similar drug-induced delta-delta QT changes were reported in published TQT studies [7]–[9]. Based on these observations, we conclude that QT_RMS_ and RT_PK_ intervals lengthen in response to moxifloxacin administration and that these values track changes in ventricular repolarization comparable to findings using QT_II_.

**Figure 8:**
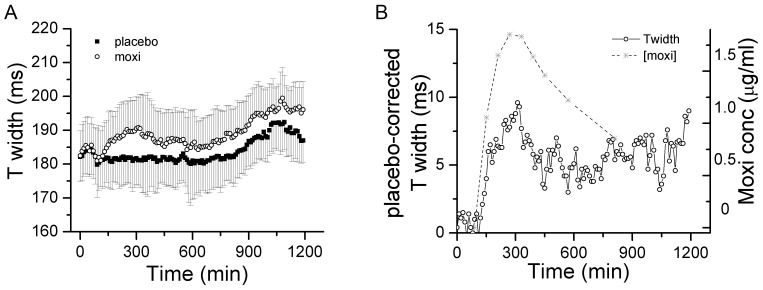
Dispersion of repolarization as measured by RMS T wave width. **Panel A**, The time course of changes in patient specific RMS ECG T wave width, TW, is presented. Data points represent 10 min averages (mean ± STD) from 68 subjects following placebo (filled square) or moxifloxacin (open circles) administration. **Panel B**, T width values during placebo treatment arm were subtracted from corresponding values during moxifloxacin treatment to obtain placebo-corrected values and plotted together with mean moxifloxacin plasma concentrations. The time course of changes in placebo-corrected T width correlated with changes in plasma moxifloxacin levels.

The width of the RMS T wave provides a robust estimate of the range of ventricular repolarization times and thus is a reflection of the dispersion of repolarization [2]. TW is an order of magnitude less rate dependent than QT_RMS_ or RT_PK_ and hence was not rate corrected [2]. The time course of changes in TW during the study periods is plotted in Fig. 8. Moxifloxacin resulted in TW widening that followed a time course similar to prolongation of QT_RMS_, RT_PK_ and QT_II_ intervals. Placebo-corrected TW lengthening correlated with moxifloxacin plasma levels and reached its maximum change of 9 ms around 3 hours after moxifloxacin administration.

### Application of RMS electrocardiography in congenital LQTS

**Figure 9:**
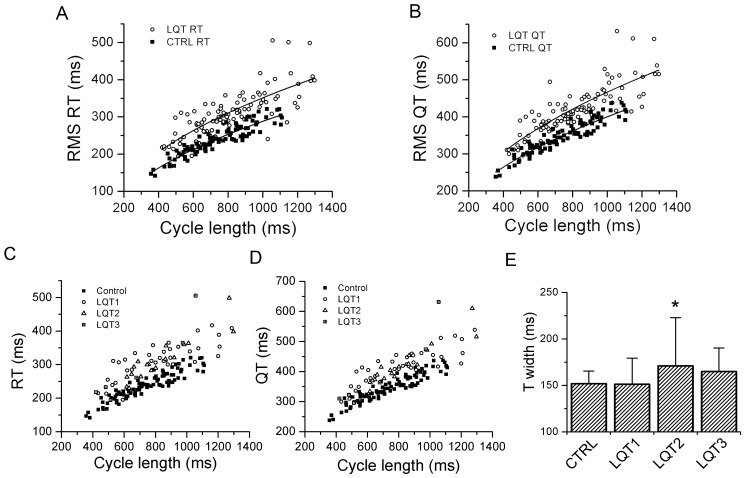
QT_RMS_ and RT_PK_ relationships in LQTS children and age-matched controls. **Panels A and B**, QT_RMS_ and RT_PK_ values from 12 lead ECGs are plotted versus corresponding cycle lengths for LQTS children (open circles) and age-matched controls (closed squares). Solid lines represent a parabolic fit to the data. **Panels C and D**, RMS ECG parameters by LQTS genotype. RT_PK_ and QT_RMS_ values obtained from 12 lead ECGs are plotted versus corresponding cycle lengths for healthy control subjects and LQTS subtypes 1–3. **Panel E**, Comparison of RMS T width for healthy control subjects and the most common LQTS subtypes. Data represent mean ± STD. ^*^ p<0.00005, ANOVA).

After determining the validity and precision of RMS ECG intervals to track changes in drug-induced ventricular repolarization in adult subjects, we turned our attention to children with congenital LQTS. The RMS signal was derived from 12-lead ECGs obtained in 97 children with congenital LQTS and 97 age-matched, healthy controls. The relationships between QT_RMS_ and RT_PK_ intervals versus CL are represented in Fig. 9 (A and B). Similar to the moxifloxacin group, the relationships between QT_RMS_ and RT_PK_ versus cycle length were shifted upwards for the LQTS group compared to placebo (p<0.05). We were unable to derive patient-specific correction factors due to the short duration of the standard 12-lead ECG. Applying the Fridericia correction (α = 0.33) was only modestly successful in correcting the QT_RMS_ and RT_PK_ intervals (data not shown).

The distribution of QT_RMS_, RT_PK_ and TW intervals by individual LQTS genotype are presented in Fig. 9C, D and E. The distributions of the QT_RMS_ and RT_PK_ intervals were generally similar for LQT1, LQT2 and LQT3 subjects (Fig. 9 C and D). A comparison of RMS TW measurements for the control and LQTS subjects is presented in Fig. 9E. RMS TW measurements for the LQT2 subgroup were significantly longer than control subjects or patients with LQT1 and LQT3 (p<0.00005, ANOVA). Based on these observations, we conclude that the RT_PK_, QT_RMS_ and RMS TW measurements appropriately track changes in ventricular repolarization in children with congenital LQTS.

**Figure 10:**
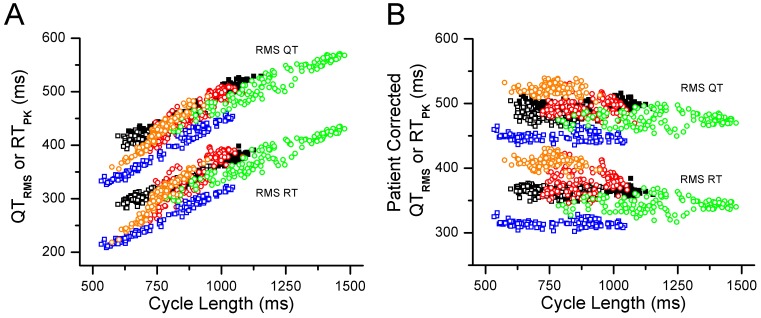
Measured and patient-specific rate corrected QT_RMS_ and RT_PK_ derived from 24-hour. Holter recordings in a subset of pediatric LQTS subjects. **Panel A**, The mean 10 min averaged QT_RMS_ and RT_PK_ intervals for 7 LQTS subjects are plotted versus corresponding cycle length, highlighting a mostly linear relationship. **Panel B**, QT_RMS_ and RT_PK_ values were corrected for heart rate using a patient-specific linear fit to the QT_RMS_ and RT_PK_ versus cycle length relationship. Similar to data obtained in adult subjects, the patient-specific heart rate corrected QT_RMS_ and RT_PK_ intervals did not vary with cycle length.

Because we were unable to calculate patient-specific heart rate corrections for the RMS ECG intervals of the standard 12-lead ECG, we derived RMS ECG signals from high-resolution 24-hour Holter monitor recordings in 7 pediatric LQTS subjects (2 LQT-1, 2 LQT-2, 2 LQT-5 and 1 LQT-3 patients). Similar to the adult Holter data, 10-minute averages of QT_RMS_ and RT_PK_ intervals correlated with CL in a linear fashion (Fig. 10A). Applying a linear patient-specific heart rate correction resulted in corrected values that varied little with CL (Fig. 10B).

## Discussion

The rate-corrected QT interval measured from a 12-lead ECG (using lead II) is the clinical standard for the non-invasive assessment of ventricular repolarization, yet these measurements and their interpretation are problematic. The QT measurement itself has a low signal-to-noise ratio, thereby complicating the precise determination of end of the T wave, especially in presence of encroaching P or U waves. The encroachment of the P wave onto the T wave downslope poses a particular problem for the measurement of the QT interval in newborn children with rapid heart rates. In this context, we tested the validity of RMS electrocardiography to detect changes in ventricular repolarization in the setting of drug-induced and congenital LQTS.

Initial animal studies detailed the cellular and organ level basis and rationale for the RMS ECG measurements, specifically the RT_PK_ and TW intervals [1]–[5]. The RT_PK_ is highly and linearly correlated with mean ventricular activation-recovery intervals as measured from unipolar epicardial electrograms [3], [4]. The activation-recovery interval correlates with transmembrane ventricular action potential duration, as measured with floating electrodes in control conditions and during local alterations in repolarization [3], [4]. Thus, the RT_PK_ interval provides an estimate of mean ventricular action potential duration. By contrast, the QT interval does not provide a similar correlate of action potential duration. Rather, the QT is an estimate of the interval between the earliest depolarization and the latest repolarization in the measured lead. The width of the RMS T wave corresponds to the range of ventricular repolarization times and thus is a measure of the dispersion of repolarization [2]–[5], which is an established factor contributing to arrhythmia vulnerability.

While the animal data verifying the relationship between RMS ECG intervals and cellular electrophysiology is compelling, the application of RMS electrocardiography to human subjects is limited [11], [12]. Thus, the primary goal of this study was to validate the use and test the precision of RMS electrocardiography to detect prolongation of ventricular repolarization in the setting of drug-induced and congenital LQTS in human subjects. We used data from a TQT study that provided a highly controlled and regulated environment to determine changes in RMS ECG repolarization features in response to randomized and blinded administration of placebo and moxifloxacin. Moxifloxacin is the industry “gold-standard” control in that multiple studies have shown a ∼10 ms prolongation in QTc interval in healthy control subjects. Likewise, we found that both RT_PK_ and QT_RMS_ lengthened in response to moxifloxacin administration, based on raw and CL-corrected values. Similar to other published TQT studies [7]–[9], the peak placebo-corrected RT_PK_ and QT_RMS_ intervals correlated with the peak moxifloxacin plasma levels. The Bland-Altman analysis confirmed that the standard-of-care lead II QT tangent method and the QT_RMS_ are equivalent measures of repolarization duration. Importantly, the variance of the QT_II_ was substantially *larger* than that of the QT_RMS_ and RT_PK_, underscoring the better precision of the RMS based measures. A comparison of the STD of the RMS measures to those of other published QT studies (range 6–18 ms, Table 3 in [8]) also confirms the better precision of RMS electrocardiography to detect significant changes in repolarization. Accuracy and precision are critical in determining the number of subjects necessary to detect a significant difference in repolarization in a TQT study and thus the cost of adhering to FDA requirements. Interestingly, the width of the RMS T wave increased following moxifloxacin administration and the peak, placebo-corrected changes in T width correlated with the peak moxifloxacin plasma levels. Taken together, these data suggest that moxifloxacin prolonged ventricular repolarization and increased the dispersion of repolarization in healthy subjects.

At a fundamental level, action potential duration and all ECG estimates of ventricular repolarization vary with heart rate in a complex and patient-specific manner [13]. Moreover, ECG estimates of repolarization adapt differently to increasing or decreasing heart rates, a process termed RR/QT hysteresis [14]. We were surprised to find that 10 min averages of QT_RMS_ and RT_PK_ intervals correlated with CL in a linear fashion. Such a linear relationship is not true for QT intervals measured from consecutive ten-second, 12-lead ECGs [13]. The goal of our study was not to investigate the fundamental nature of repolarization hysteresis. However, we speculate that by averaging the QT_RMS_ and RT_PK_ intervals over a 10-minute period, the short-term dynamics of repolarization hysteresis were smoothed out, such that the relationship between these intervals and CL was mostly linear.

In addition to detecting drug-induced changes in ventricular repolarization, RMS electrocardiography was also successful in tracking prolonged ventricular repolarization in the setting of congenital LQTS. Both the QT_RMS_ and RT_PK_ intervals were prolonged in LQTS children compared to controls. This study corroborates earlier animal studies regarding the ability and precision of RMS electrocardiography to detect changes in ventricular repolarization and supports the utility of RMS electrocardiography as a novel measure of repolarization in humans.

The RMS TW measurements for children with LQT-2 were significantly longer than other LQTS subtypes or healthy controls. This finding is consistent with the RMS TW lengthening observed during moxifloxacin administration. Mechanistically, moxifloxacin treatment and LQT-2 patients share a common pathophysiology in that moxifloxacin blocks the hERG potassium channel [15] and LQT2 patients have mutations in the gene encoding the hERG channel [16], [17]. The implication is that hERG channel dysfunction prolongs the dispersion of repolarization. It is not clear why dysfunction of the other primary delayed rectifier potassium current, *I*_Ks_ (LQT-1) did not also increase the dispersion of repolarization as measured by TW.

## Study limitations

TQT studies are performed in highly controlled research environments in order to minimize day-day differences and facilitate direct comparisons between placebo, moxifloxacin or study drug treatments. While RMS ECG-derived measurements nicely track drug-induced changes in ventricular repolarization in the TQT environment, the applicability of RMS ECG measurements to the “real world” environment was not fully addressed in the current study. However, in our limited Holter analysis of pediatric patients with LQTS, we were able to calculate patient-specific heart rate corrected values even while the children went about their normal active behaviors.

Another limitation of the study is that we did not have access to the QT measurements reported to the FDA and thus were unable to compare our QT_RMS_ and RT_PK_ measurements with annotated QT measurements reported in the TQT study. Finally, the data derived from the standard 12-lead ECG must be interpreted cautiously in light of the very short nature of data acquisition (10 seconds). While we were able to detect significant differences in RMS ECG parameters between LQTS and healthy control children, heart rate correction based on the subject group as a whole was inadequate due to the highly variable inter-subject relationships. In particular, we must be cautious when interpreting the results of differences in RMS TW between control subjects and the subtypes of LQTS, especially in light of the relatively small numbers within each subtype.

## Summary

In summary, this study builds upon earlier animal studies establishing the cellular basis for RMS ECG intervals [1]–[5] and establishes the validity of RMS electrocardiography to detect drug-induced and congenital abnormalities in ventricular repolarization in human subjects. An advantage of the RT_PK_ interval is that measurement of the peak of the RMS T wave signal is more precise and easier to measure than a low amplitude signal, such as the end of the T wave. Moreover, the RT_PK_ measurement has a specific cellular correlate in that it corresponds to the mean ventricular APD. RMS electrocardiography may be ideally suited to measure repolarization in newborn patients where the P wave typically encroaches on the end of the T wave. The inability to precisely detect the end of the T wave in newborn children is one of the major obstacles to universal screening of newborns for LQTS. By focusing on the peak of the T wave, RT_PK_ intervals may provide a reliable, robust measure of ventricular repolarization and facilitate development of ECG screening tools for early detection of LQTS.
